# Patterns of antipsychotic prescription in inpatient and outpatient psychiatric settings: a real world study

**DOI:** 10.1192/j.eurpsy.2024.1187

**Published:** 2024-08-27

**Authors:** T. G. Prodi, E. Piccoli, C. Bucca, A. Tomasoni, B. Dell’Osso

**Affiliations:** ^1^University of Milan, Department of Mental Health, Department of Biomedical and Clinical Sciences “Luigi Sacco”; ^2^University of Milan, Aldo Ravelli” Center for Nanotechnology and Neurostimulation, Milan, Italy; ^3^Stanford University, Department of Psychiatry and Behavioral Sciences, Stanford, United States; ^4^University of Milan, Centro per lo studio dei meccanismi molecolari alla base delle patologie neuro-psico-geriatriche, Milan, Italy

## Abstract

**Introduction:**

Antipsychotic (AP) are increasingly prescribed off-label and used as, but not limited to, anti-aggressive, anti-impulsive, and anti-suicidal medication. The use of second-generation AP (SGAs) has progressively increased compared to first-generation AP (FGAs). FGAs cause more extrapyramidal motor side effects and tardive dyskinesia than SGAs, whereas SGAs generally cause more weight gain and cardiometabolic adverse effects.

**Objectives:**

Aim of this observational study was to describe the socio-demographic and clinical features of the patients receiving new AP treatment and the features of the pharmacological treatment itself in “real world” context. Furthermore, we aimed to compare socio-demographic and clinical characteristics of the subjects who were prescribed either FGAs or SGAs.

**Methods:**

Data were collected on the latest new AP prescriptions issued across different settings (two psychiatric wards; five outpatients clinics; and one rehabilitation community) belonging to ASST Fatebenefratelli Sacco (located in Milan) in reverse chronological order from May 2023.

Socio-demographic and clinical variables of the subjects who received new AP treatment were collected through medical records. We compared age, age at onset, age at first pharmacological treatment, duration of illness, duration of untreated illness, treatment duration, number of hospitalization and admissions to Day Hospital services, involuntary commitments and suicidal attempts in patients who received either FGAs or SGAs. Chi-square was used for qualitative variables and t-test for quantitative variables. Data were collected anonymously and analyzed using SPSS v.27.

**Results:**

The sample included 155 new AP prescriptions, out of which 29.2% were formulated in the psychiatric wards, 66.9% in the outpatient clinics e 3.9% in the rehabilitative community. Mean age of the subjects was 41.1 ± 16.9 years, 53.2% were male.

The most represented diagnoses were psychotic disorders (32.2%), personality disorders (24.8%), bipolar disorder (16.1%) and depressive disorder (12.8%).

90.7% of new AP prescriptions were SGAs. The most prescribed were aripiprazole (30.5%), quetiapine (21.2%) and olanzapine (15.2%); while the most prescribed FGAs were haloperidol (5.3%), zuclopenthixol (2%) and chlorpromazine (1.3%). 26.2% of the prescriptions were in monotherapy and 83.8% were for oral administration.

The reasons for introduction were partial or absent response to previous treatments (52.3%), disease onset (23.5%), non-compliance (8.3%), adverse effects to previous treatments (6.8%) or other (9.1%). Patients treated with FGA had a longer duration of untreated illness (p<0,001) and a greater number of lifetime hospitalizations (p<0,001) and involuntary commitments (p=0,002).

**Conclusions:**

Patients treated with SGAs have a shorter duration of untreated illness and also lower chance of lifetime hospitalization and involuntary commitment.

**Disclosure of Interest:**

None Declared

